# Colour matters more than shape for chimpanzees’ recognition of developmental face changes

**DOI:** 10.1038/s41598-020-75284-2

**Published:** 2020-10-23

**Authors:** Yuri Kawaguchi, Koyo Nakamura, Masaki Tomonaga

**Affiliations:** 1 Primate Research Institute, Kyoto University, Aichi, Japan; 2 Japan Society for the Promotion of Science (JSPS), Tokyo, Japan; 3 Faculty of Science and Engineering, Waseda University, Tokyo, Japan; 4 Keio Advanced Research Centers, Tokyo, Japan

**Keywords:** Evolution, Psychology

## Abstract

Social primates must recognise developmental stages of other conspecifics in order to behave appropriately. Infant faces have peculiar morphological characteristics—relatively large eyes, a small nose, and small mouth—known as baby schema. In addition, the infant faces of many primate species have unique skin coloration. However, it is unclear which features serve as critical cues for chimpanzees to recognise developmental changes in their faces. The present study aimed to investigate the relative contributions of facial shape and colour to age categorisation in chimpanzees. We used a symbolic matching-to-sample task in which chimpanzees were trained to discriminate between adult and infant faces. Then, we tested how their age category judgments transferred to a series of morphed faces which systematically differed in facial shape and colour. Statistical image quantification analysis revealed significant differences both in shape and colour between adult and infant faces. However, we found that facial coloration contributed to age categorisation in chimpanzees more than facial shape. Our results showed that chimpanzees use unique infantile facial coloration as a salient cue when discriminating between adult and infant faces. The display of their developmental stages through facial colour may help chimpanzees to induce appropriate behaviour from other individuals.

## Introduction

Faces are a rich source of biological and social information such as age, identity, gender, emotional states, impressions and so on in humans^1,2^. The ability to extract various information from faces plays a critical role in a wide range of social and emotional interactions. Comparative cognitive studies have been investigated the role of face perception across species in order to reveal the evolutionary origins of face perception in humans. Thus, non-human primates are a good target because of their phylogenetic closeness to humans. Most non-human primates have trichromacy like humans, which is supposed to benefit them for detecting variations in facial coloration indicating such as emotional, breeding, or health status^3–6^. Indeed, non-human primates are able to extract different types of information from faces^7^, including facial identity^8^, facial expressions^9,10^, sex of the face^11^, and so on, partially due to high sensitivity to facial coloration.

Among various social traits, age perception is a vitally important facial attribute because this particular cue helps animals behave appropriately toward other animals^12^. Animals should not treat an adult animal like an infant and vice versa. For example, adult animals should avoid immature animals as a mating partner; unfamiliar individuals can be a threat if s/he is an adult but probably not if s/he is an infant. Although non-human animals can use various physical cues when judging age, including body size, body movement and vocalisation, one prominent facial cue to age perception is baby schema (*Kindchenschema*)^13^. Baby schema is a set of morphological features that are characteristic of infant appearance such as larger eyes relative to head size, a high and protruding forehead, and a smaller nose and mouth^13^. Studies have shown that the baby schema makes human faces appear cuter and enhances viewers’ caretaking motivations^14–19^. Researchers have premised that baby schema features are shared across various species^13,19^. While baby schema is supposed to exist in various species, many species of primate also display ‘infantile coloration’, or unique skin and/or coat coloration, during infancy^20–24^. For example, in some colobine species, infants have a bright fur coat while their mothers have a dark coat^21^. One plausible functional role of these colorations is to trigger care-taking behaviours in adults. Studies have shown that affiliative behaviours are most often observed toward infants displaying infantile coloration, and decrease as infants age and lose their infantile coloration^20^. Taken together, both facial shape and colour properties can provide critical information related to age, at least in some primate species.

These infantile features are assumed to affect recognition in non-human primates, but it remains unclear whether non-human primates are sensitive to these features for extracting age information. A few studies have reported non-human primates’ visual preference for infants. For example, an empirical study with eye-tracking demonstrated that adult chimpanzees (*Pan troglodytes verus*) prefer to look at infant faces when presented with the pictures of conspecific mothers and infants^25^. However, this preference disappeared when the chimpanzees were presented with grey-scaled images of faces, controlling for face brightness. This suggests that unique infant face colour plays an important role in chimpanzees. Visual preferences for infants were also found in Japanese macaques (*Macaca fuscata*) and Campbell's monkeys (*Cercopithecus campbelli*)^26^. Furthermore, preference for natal colour has observed in rhesus macaques (*Macaca mulatta*) in one study^27^, but not in the other^28^. Higley et al.,^27^ investigated macaques’ approach behaviour to conspecific infants whose face and/or fur was dyed and reported their attraction to neonatal-like facial skin coloration but not others. On the other hand, Gerald et al.,^28^ also tested the role of neonatal colour in the same species by presenting digitally colour-manipulated face images but found no significant effect of colour on rhesus macaques’ behaviour. Therefore, the evidence on visual preference for natal colour is still controversial and has been tested only in limited species.

Although the literatures indicate that non-human primates somehow discriminate the age of conspecifics from their appearance, there is little direct evidence that primates are able to explicitly recognise age from faces. It is only recently that humans’ ability to distinguish infants from adults by their faces was reported in non-human primates: Kawaguchi et al.^29^ trained capuchin monkeys to discriminate between grey-scaled facial images of adults and infants using a symbolic matching-to-sample task and showed that capuchin monkeys can successfully discern adult and infant of human and conspecific faces. Given that the facial images were greyscale, the capuchin monkeys must have extracted facial information besides coloration to discern the age categories of faces.

This begs the question: what kind of facial features are critical to non-human primates’ ability to recognise the age of another face? Recent advances in image-processing technology enable researchers to identify which features are important for recognising various facial attributes^30^. Humans perceive various facial attributes through a combination of facial shape and colour properties^1^. Studies on human face perception have investigated how facial shape (e.g., configuration or size of facial parts) and surface (e.g. colour or texture) features contribute to age perception. It is shown that perception of facial skin surfaces is a more important cue than facial shape in human age perception: Burt and Perrett^30^ manipulated both facial shape and colour separately and found colour had a greater effect than shape on age perception, and Lai et al.^31^ investigated the relative contribution of facial shape and colour on age perception and found that texture made greater contribution than shape to age perception.

Although comparative cognitive studies have also studied the role of facial shape^32^ and colour cues^33,34^ in the perceptions of facial attributes, the morphing techniques used to manipulate faces’ morphology and surface have seldom been applied to non-human primates, with few exceptions. For example, Koba et al.^11^ investigated the role of facial shape on sex discrimination in Japanese macaques. In the study, two Japanese macaques were trained to discriminate between male and female faces. Then, morphed facial stimuli (e.g. a male face with female morphology) were tested and the authors found that the macaques used both morphological and non-morphological cues to discern the sex of the faces. However, they did not address which set of cues was more important for sex discrimination. Therefore, to the best of our knowledge, no study has yet investigated the relative contribution of facial shape and colour to facial attributions, especially age perception in non-human primates.

In light of the above, the present study aims to test which aspects of facial features (i.e. facial shape and colour) act as cues to age perception via a symbolic matching-to-sample task. Based on previous research which shows that chimpanzees pay attention to infantile facial coloration^25^, we predicted that the chimpanzees in our study would use facial colour as a cue to discern the age category of a face, but did not know whether they would use face shape in the same way. We first trained chimpanzees to discriminate between average adult and infant faces and then tested how they responded to morphed faces with different levels of facial shape and colour ranging from adult to infant faces. By using morphed faces, we were able to have systematic control over facial shape and colour information and eliminate irreverent information which occurs and varies randomly across individual facial photographs (e.g. lighting condition). Besides Koba et al.^11^, some other studies have used synthesised face stimuli such as a morphed or “enhanced” face to study facial recognition in non-human primates^35–37^. By testing chimpanzees’ responses to morphed stimuli, this study attempts to determine how important each facial feature is for chimpanzees’ ability to recognise age information in other faces.

## Methods

### Participants

Five adult female and one male chimpanzees (aged 18–42 years) participated in the experiment (see Table 1). They lived in Kyoto University Primate Research Institute as a social group of 11 individuals. Two of them have given birth before. All the participants were already familiar with cognitive tasks involving touch panels, including matching-to-sample tasks with face stimuli^38,39^. One chimpanzee, Ai, used to be trained to use lexigrams for ape-language project (called “Ai Project”)^40,41^. She was able to label 67 words representing objects^42^, colour^42,43^, number^44^, individual^41^ and personal pronouns^45^. Of the six chimpanzees, three dropped out of the study because they could not pass the criteria set during the very beginning of the training (see Table 1 and Supplementary Note for more details). Data from the three remaining chimpanzees is analysed below. The chimpanzees were housed in an enriched environment which featured both an indoor enclosure and an outdoor compound and vegetation^46^, had free access to water, and received food (fresh fruits, vegetables, sweet potatoes, and nutritionally balanced biscuits) several times each day. All research procedures followed institutional guidelines (Primate Research Institute 2010 version of ‘The Guidelines for the Care and Use of Laboratory Primates’) and the experimental protocol was approved by Animal Welfare and Animal Care Committee of the Primate Research Institute (2019-064) and the Animal Research Committee of Kyoto University.

**Table 1:** Participant information.

Individual name (GAIN ID^a^)	Sex	Age	Birth experience	Training
Ai (0434)	Female	42	Parous	Passed
Chloe (0441)	Female	38	Parous	Passed
Pal (0611)	Female	19	Nulliparous	Passed
Cleo (0690)	Female	19	Nulliparous	Dropout
Pendesa (0095)	Female	42	Nulliparous	Dropout
Ayumu (0608)	Male	19	–	Dropout

^a^Individual information based on the database of GAIN (Great Ape Information Network).

### Apparatus

The experiment was conducted in an experimental booth (1.8 m × 2.15 m × 1.75 m). Each chimpanzee voluntarily came to the experimental booth through a corridor connected to the living areas. A 17-inch touch-sensitive LCD monitor (IO Data LCD-AD172F2-T, 1280 × 1024 pixels) and universal feeder (Biomedica, BFU310-P100) were installed in the booth. We controlled stimulus presentation, response detection, and food delivery by a customised program written in Microsoft Visual Basic 2010 Express working on a personal computer (CPU: Core [TM] i3-4130 3.40 GHz; Intel, Santa Clara, CA).

### Stimuli

**Figure 1:**
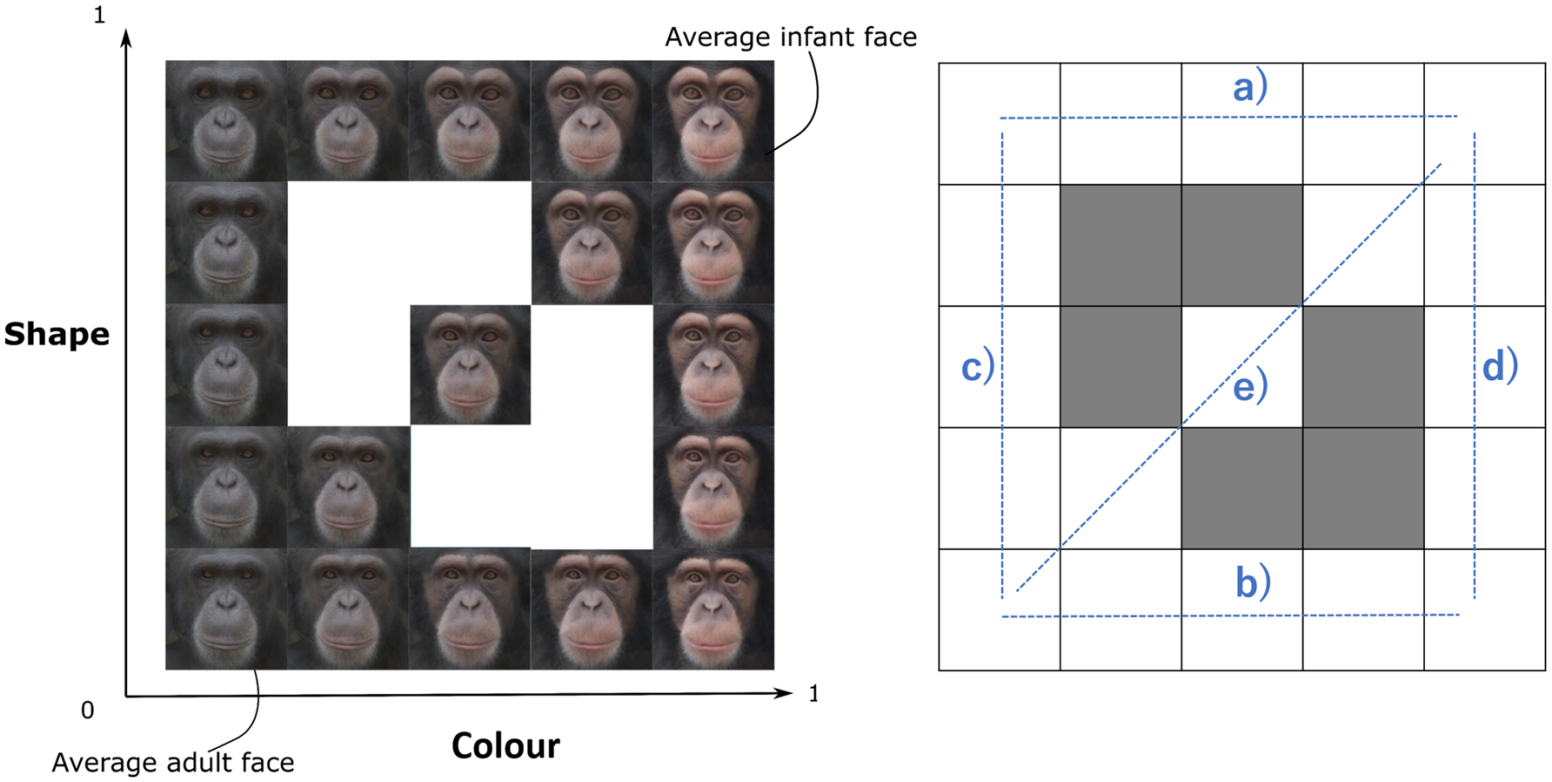
The morphed face stimuli used in test phase: (**a**) faces with varying facial colours between adult and infant faces with infant facial shapes, (**b**) faces with varying facial colours between adult and infant faces with adult facial shapes, (**c**) faces with varying facial shapes between adult and infant faces with adult facial colours, (**d**) faces with varying facial shapes between adult and infant faces with infant facial colours, and (**e**) faces with co-varying facial shapes and colours. The facial images were generated with Psychomorph software (version 6; https://users.aber.ac.uk/bpt/jpsychomorph/).

First, adult and infant chimpanzee faces were averaged across eight individuals (half male, half female). All the pictures were taken by one of the authors (YK) or provided by her colleagues. Photographed infants were 2–10 months old and adults were 13–22 years old at the time the pictures were taken. All facial images were scaled and realigned based on eye size and position. To delineate the features of each face, 118 landmarks were placed for each facial image (see Supplementary Figure S1) with Psychomorph software^47^, which has been widely used to morph chimpanzee, capuchin monkey, rhesus macaque, and human faces^32,35,37,48^. To average adult and infant face images, we superimposed the adult facial images on one another and the infant facial images on one another. We then morphed the shape and colour of these averaged faces. The shape and colour of each morphed face independently varied between 0 and 1 (where 0 represents adult features and 1 represents infant features), resulting in 19 morphed faces as illustrated in Fig. 1. Specifically, these 19 faces consisted of: (a) faces with varying facial colours between adult and infant faces with infant facial shapes, (b) faces with varying facial colours between adult and infant faces with adult facial shapes, (c) faces with varying facial shapes between adult and infant faces with adult facial colours, (d) faces with varying facial shapes between adult and infant faces with infant facial colours, and (e) faces with co-varying facial shapes and colours.

To generate training stimuli, we randomly chose three adult faces from a pool of eight adult faces and then created 10 different “three-morphed average adult faces” (see Supplementary Figure S2). We also created 10 “three-morphed infant average faces” in the same manner. A total of 20 adult and infant average faces were used in the training phase and 19 morphed faces were used in the test phase. Face contours, including ears, did not appear in any of the stimuli since the boundary between face and body was not always obvious in the original pictures.

Two geometric figures (180 × 180 pixels) were prepared for the comparison stimuli. These geometric figures contained some elements which have been used in “Ai Project” for one of our participant chimpanzees, but the combinations of the elements were unfamiliar to her. One geometric figure was assigned to “adult,” and the other to “infant” and this assignment is consistent among the participants.

### Procedure

**Figure 2:**
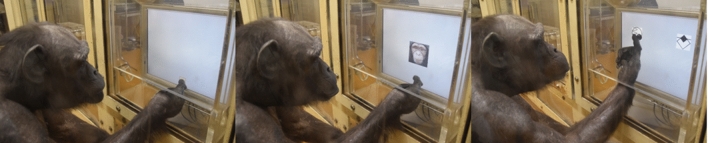
The procedure of experiment (symbolic matching to sample).

The chimpanzees were trained to discriminate between adult and infant faces in a zero-delay symbolic matching-to-sample task. In the task, a sample stimulus appeared in the centre of the monitor when a chimpanzee touched the self-start key on the bottom of the monitor. Immediately after the chimpanzee touched the sample stimulus, it disappeared and two geometric figures appeared as comparison stimuli—one at each top corner of the monitor (Fig. 2). The left–right position of the comparison was randomised within each session. When chimpanzees touched the geometric figure which corresponded with the presented image, they received a piece of apple and a chime sound played. When they chose the wrong geometric figure, a buzzer sound was played, no food reward was presented, and the same trial was repeated as a correction trial. When they made a correct response or made three mistakes, the next trial started. This procedure was almost identical to that of Kawaguchi et al.^29^.

#### Training phase

The first training session consisted of 54 trials. Training began by presenting chimpanzees with a pair of three-morphed average adult and infant faces. Adult and infant faces were presented with the same frequency in each session. If a chimpanzee achieved more than 85% accuracy in two consecutive sessions, we considered that they met the training criterion. Then, as a probe test, three new pairs (i.e. six stimuli) were presented for the subsequent two sessions to test the robustness of their ability to discern age from faces. A single probe test session consisted of 18 probe trials and 36 baseline trials. In the probe trials, each new stimulus was presented in three trials as a probe test within a session. In the baseline trials, the learned stimuli were presented. Rewards were delivered regardless of the chimpanzees’ choices in the probe trials in order to avoid specific learning for test stimuli, but only correct choices were rewarded in baseline trials. After completing two probe sessions, the chimpanzees were trained with these three pairs besides the learned pairs. The training session consisted of 30 trials with new stimuli and 28 trials with learned stimuli. The combinations of the stimuli pairs were fixed but the order of their introduction was counterbalanced across individuals (Table S1). We repeated the training sessions and probe test sessions four times so that the chimpanzees learned 10 stimuli pairs.

#### Test phase

When participants’ performance met the training criterion for the session including all 10 pairs, test sessions were carried out. A single test session consisted of 19 test trials and 40 baseline trials. Each test stimulus was presented once as the all-reinforced probe test trial in a test session. In baseline trials, training stimuli (i.e. 10 pairs of three-morphed faces) were presented in random order. Test sessions were conducted 16 times in blocks of four sessions. We confirmed the baseline performance before each test block.

### Statistical analysis

#### Statistical image quantification of adult and infant faces

To ascertain whether there are reliable visual cues to facial differences between adult and infant faces, we performed facial shape and colour analysis for the 20 faces (10 adult and 10 infant faces) used as the training stimuli. For our facial shape analysis, all the facial landmarks placed on the 20 faces were superimposed by a generalized Procrustes analysis via tpsRelw software (version 1.65). This analysis was done to standardise face size and optimise face rotation and translation to minimise the distances between corresponding landmarks. Next, we carried out a relative warp analysis (i.e., principal component analysis (PCA)) for parameter α = 0 via tpsRelw (version 1.65) to reduce dimensionality. The first five components were selected because these components accounted for more than 95% of facial shape variation. To further identify any statistically significant differences between adult and infant facial shapes, we performed Bonferroni corrected t-tests for each of the five shape principal component (PC) scores between the adult and infant faces.

For our facial colour analysis, all the facial images were unwarped to the average face configuration across the 20 faces. Next, the RGB colour intensity of the facial images was converted into a CIELAB colour space. Then, a PCA to the LAB intensity of the facial images was carried out in MATLAB (version 2019a), and the first seven components were selected because these components accounted for more than 95% of facial colour variation. To identify any statistically significant differences between adult and infant facial coloration, we performed Bonferroni corrected t-tests for each of the seven colour PC scores between the adult and infant faces.

#### Behavioural data analysis

The number of correct responses in the probe test during the training session was analysed via a two-tailed binomial test with 50% as a chance level for each time introducing new stimuli. The number of trials which were judged as ‘infant’ faces for each of the 19-test stimuli was analysed with a generalized linear mixed model (GLMM) in order to test whether shape and colour of each stimuli affected the chimpanzees’ responses to them. All statistical analyses were conducted on R 3.5.1^49^ with the “lme4” package^50^. For the GLMM analysis, we included the age judgment (infant versus adult, coded as 1 or 0) as a response variable, and the shape and the colour score of stimuli as explanatory variables. The shape and colour scores were centred by subtracting mean scores, thereby ranging from − 0.5 (average adult) to 0.5 (average infant). We also set participant, interaction between participant and shape, and interaction between participant and colour as random effects. We used a binomial distribution with a logit link function. We started from the simple model with main effects of shape and colour as an explanatory variable, and we tested whether the inclusion of the interaction effect between shape and colour improved the goodness of fit of the model by a likelihood ratio test. The likelihood ratio test showed that adding the interaction effect did not significantly improve the goodness of fit (χ^2^ = 3.2, *p* = 0.07). Therefore, we selected the simple model without interaction effect as the best-fitted model.

## Results

### Statistical image quantification of adult and infant faces

**Figure 3:**
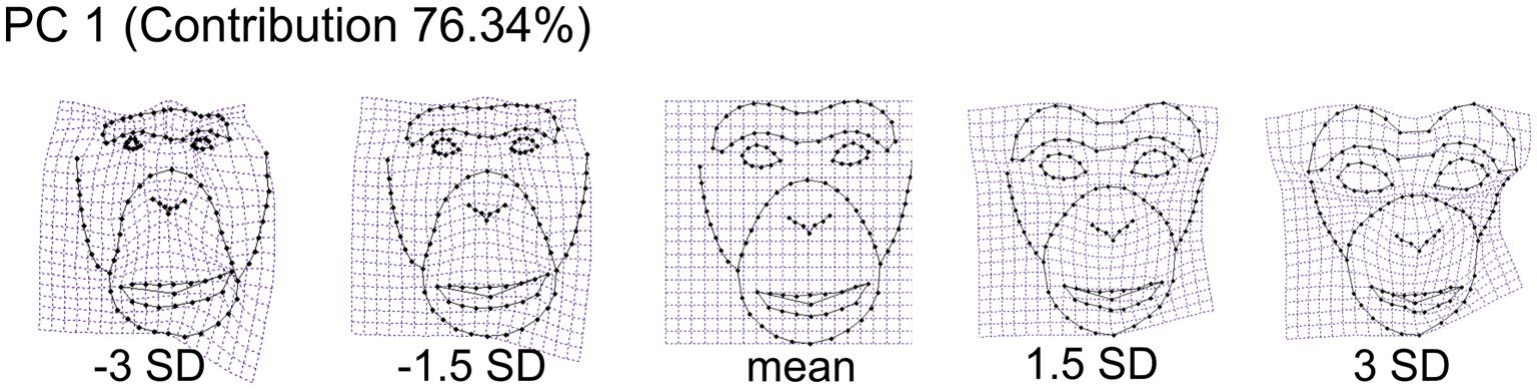
Facial shape variations along the axis of the first principal component: The theoretical values of − 3, − 1.5 SD, average, + 1.5, and + 3 SD. The facial images were generated with TpsRelw software (version 1.70; https://tpsrelw.software.informer.com/).

**Figure 4:**
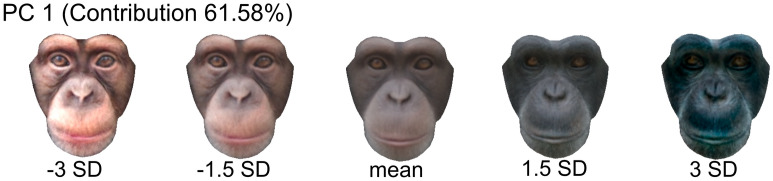
Facial colour variations along the axis of the first principal component. The theoretical values of − 3, − 1.5 SD, average, + 1.5, and + 3 SD. The facial images were generated with MATLAB (version 2018a; https://uk.mathworks.com/products/matlab.html).

To identify the facial features that differed between adult and infant faces, we performed multiple t-tests for facial shape and colour components extracted from the PCA. For the facial shape, the five components were individually tested by a t-test with a Bonferroni correction (0.05/5 = a corrected α level of 0.01). The results revealed that infant faces (average = 0.050) had significantly larger PC1 scores than adult faces (average = − 0.050) (*p* < 0.001), while there were no significant differences for any other components (all, *ps* > 0.10). Facial shape variations along PC1 are visualized in Fig. 3 (see Figure S3 for all five components). Likewise, for the facial colour, the seven components were individually tested by a t-test with a Bonferroni correction (0.05/7 = a corrected α level of 0.0071). The results revealed that infant faces (average = − 0.22) had significantly smaller PC1 scores than adult faces (average = 0.22) (*p* < 0.001), while there were no significant differences for any other components (all, *ps* > 0.10). Facial colour variations along PC1 are visualized in Fig. 4 (see Figure S4 for all seven components).

### Behavioural task results

#### Training phase

Three chimpanzees (Ai, Chloe, Pal) completed the all of the sessions. We first investigated the number of sessions each chimpanzee required to learn facial discrimination for each time new stimuli were introduced. Each chimpanzee required 20–49 sessions to learn the first stimuli pair, but required fewer (two to four) sessions as the last new stimuli were introduced (see Supplementary data). Their response accuracy for the unfamiliar face stimuli in the probe test trials during the training phase was significantly above the chance level (*p* < 0.05) each time introducing from the beginning in all three chimpanzees (see Supplementary data). Visual inspection of the results revealed that Ai, a chimpanzee who had previous language training required slightly fewer sessions to reach the learning criteria than other two chimpanzees, but her performance for unfamiliar stimuli was not considerably different from others.

#### Test phase

**Figure 5:**
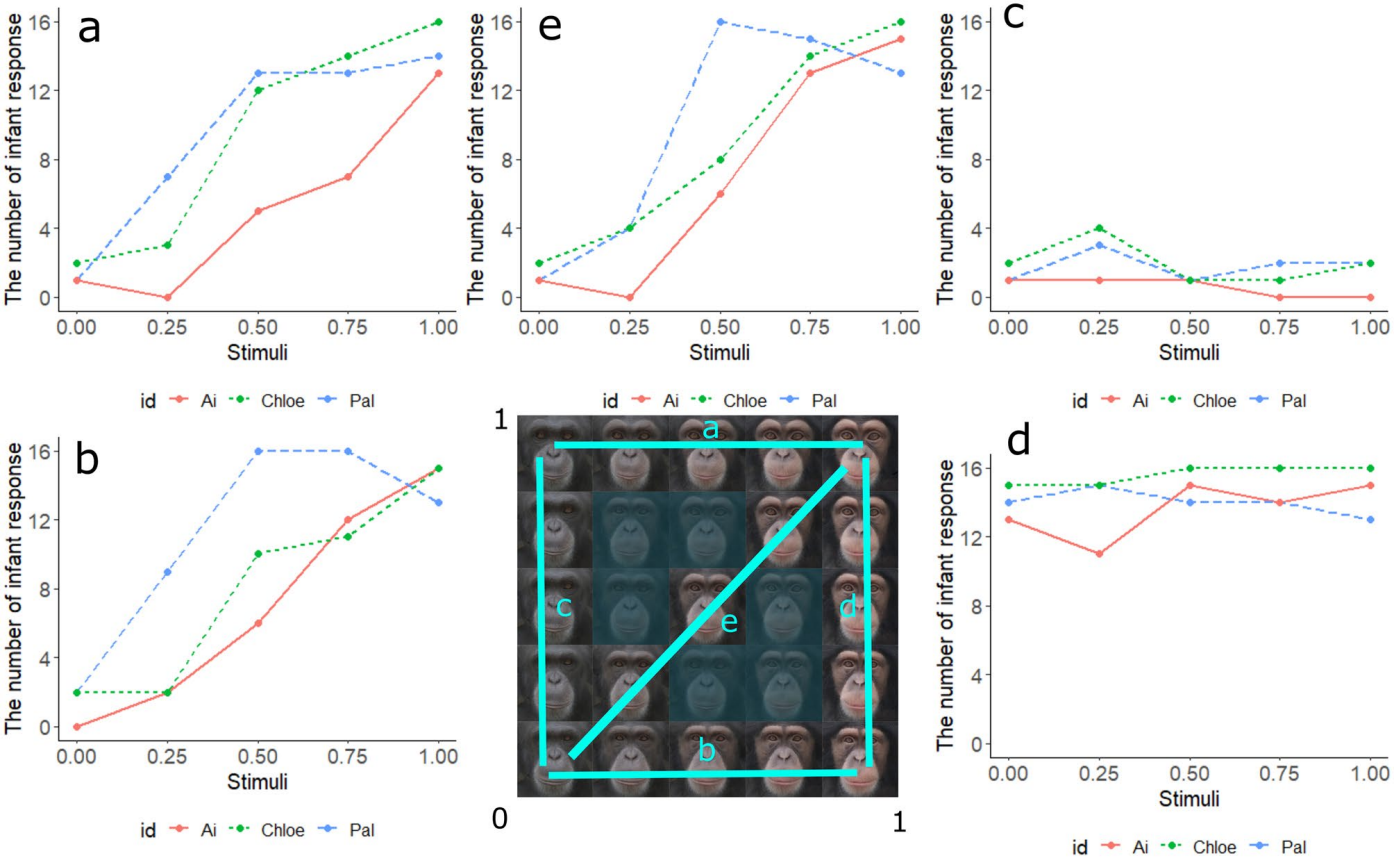
The number of infant responses for each stimulus in 16 trials. (**a**) Colour effect on *infant* shape, (**b**) Colour effect on *adult* shape, (**c**) Shape effect on *adult* colour, (**d**) Shape effect on *infant* colour, (**e**) Colour and shape effect.

Figure 5 illustrates the number of responses judged as ‘infant’ for each test stimulus in the test phase. We analysed the number of ‘infant’ responses with GLMM. The best-fitted model included the main effects of shape and colour score, but not the interaction effect between shape and colour scores. The GLMM analysis showed the significant main effect of shape (*β* = 0.66, *z* = 2.79, *p* = 0.005, 95% confidence interval (CI) [0.19, 1.12]), and significant effect of colour (*β* = 5.08, *z* = 10.60, *p* < 0.001, 95% CI [4.14, 6.02]; Table 2). The odds ratio (OR) of the main effect of shape was 1.92 (95% CI[–1.21–3.07]) while that of colour was 160.93 (60.31–471.60), suggesting that the OR of colour was 83 times bigger than that of shape in the chimpanzees’ responses. Again, visual inspection of the results found no considerable difference between Ai and the other two chimpanzees.

**Table 2:** The results of the GLMM analysis.

Factor	Estimate	SE	*Z*	*p*	95% confidence interval
Intercept	0.11	0.40	0.28	0.78	[− 0.67, 0.89]
Colour	5.08	0.48	10.60	< .001	[4.14, 6.02]
Shape	0.66	0.24	2.19	0.005	[0.19, 1.12]

## Discussion

The present study aimed to investigate the relative contributions of facial shape and colour to chimpanzees’ ability to discern age category from facial images by using a series of morphed face stimuli that differ in facial shape and colour. The results indicate that both facial shape and colour are critical cues to chimpanzees’ age judgments, but facial colour has a greater impact on these judgements than shape. These results are consistent with the results of the previous study^25^, which found that chimpanzees allocate more attention to unique infant face coloration. Our results further demonstrated that facial colour contributes more than facial shape on discriminating adult and infant faces. It is of note that at least three chimpanzees learned to discriminate adult and infant faces and were able to generalize the categorisation to unfamiliar stimuli in the training phase. These results indicate that chimpanzees can visually dissociate adults and infants from facial appearance.

Statistical quantification of the facial images revealed that there are critical differences in the shape and colour of adult and infant chimpanzees’ faces. Image analysis of facial shapes revealed the first principal component (PC 1) significantly differed between adult and infant faces. As illustrated in Fig. 3, the positive PC1 score (i.e. more infantile faces) was characterized by a baby schematic configuration, such as bigger eyes located in a relatively lower position on the face. In addition, infant chimpanzee faces have a curved supraorbital torus but adult chimpanzee faces do not. Meanwhile, the image analysis of facial colour also revealed that PC1 scores significantly differed between adult and infant faces. As illustrated in Fig. 4, the negative PC1 score was characterized by an infantile coloration, such as brighter skin colour.

As far as we know, we are the first to quantify developmental changes in facial surface in chimpanzees. Although a number of studies have focused on the cognitive processing of infantile shape features (i.e. baby schema) in human faces^14–19,30,51–53^ and non-human animal faces^19,53,54^, each of these studies have restricted themselves to human perception of facial features. Moreover, roles of infantile coloration, or species-specific infantile face features have received less attention and only been tested in rhesus macaques^27,28^. Our data showed the importance of taking species-specific features into account when attempting to determine how non-human primates discern age from faces. Taken together with this study’s findings, we can conclude that infant chimpanzees’ faces have species-general infantile features characterised by the baby schema, and that they also have species-specific infantile features such as a unique supraorbital torus or face coloration.

The results of our behavioural task showed that chimpanzees are able to differentiate between adult and infant faces. This finding is consistent with previous studies of capuchin monkeys^29^, where monkeys learned to discriminate adult and infant faces of conspecifics. This study indicated that chimpanzees pay attention to infantile face coloration and use it as a cue to differentiate between age categories of faces. Of particular interest is that not only colour cues, but also shape cues existed in chimpanzee faces for age categorisation but colour had greater effect than shape in this regard. This might be because chimpanzees are attuned to species-specific facial coloration which has been uniquely acquired (or retained) through their evolution. We cannot conclude why the effect of colour cue was more dominant than that of shape from this experiment. Facial colour can be more prominent and reliable cues than shape in their living environment when chimpanzees need to extract age information from other individuals with some distance. At the same time, it is also known that chimpanzees have some facial skin colour variations even among adults and that they likely use these to recognise and distinguish between individuals^55^. Chimpanzees may be particularly sensitive to an individual’s facial coloration in general because it tells them a lot about the individual in question.

Then, what does facial coloration tell chimpanzees about the age of a given face? Infantile facial coloration lasts into adolescence to some extent and takes longer to fade into adult colouring in chimpanzees than it does in many other primate species which also have infantile coloration. Given the nature, they may be able to get not only rough information from faces on whether the individual is infant or adult, but more precise information such as specific age class (e.g. late infancy) s/he belongs to. Another study revealed that the face coloration of Borneo orangutans (*Pongo pygmaeus*) change as they age until around 10 years old^56^. This indicates that face coloration may turn out to be a reliable indicator of developmental stage in non-human primates. Kuze et al.^56^ pointed out that infants and juveniles may enjoy tolerance from other individuals in social context thanks to this age signal. This may be also true for chimpanzees; displaying their age class by facial colour may benefit not only chimpanzee infants, but also juveniles in certain social contexts such as mating or group transfer, although it is outside the scope of this study. There have been many debates on the general function of infantile coloration such as inducing alloparenting, preventing aggression, hiding paternity and so on^20–24^. The display of their developmental stages through facial colour may help chimpanzees to induce contextually appropriate behaviour from other individuals. Nevertheless, the clear and precise function of infantile coloration remains an open question.

This study has a few limitations. First, we acknowledge that our sample size was quite small because of the difficulty of the task employed in the study (i.e. zero-delayed symbolic matching-to-sample). The sample size was as small as that in similar demanding tasks in previous non-human primate studies (e.g. two Japanese macaques^11^, three chimpanzees^57^). Given that the results among our three individuals were consistent, however, it is likely that our results reflect robust tendencies. Second, we could not conclude whether chimpanzees performed age categorisation based on something like the age category concept or merely by combining low-level visual properties of the stimuli. It could be possible to address this point by testing generalisations to other type of stimuli (e.g. whole body images or vocalizations of adults and infants). Even if their discrimination was not based on some concept of age, this would not alter the fact that they successfully differentiated between adult and infant faces and likely use the same features in their real life because such features are salient to them.

Our study showed that it is feasible to study face recognition in non-human primates in a similar manner to human by using well-controlled face stimuli, namely morphed faces to dissociate the effect of facial features such as shape and colour. Dissociating the effects of facial shape and colour on facial attributes has been a predominant method in face studies since it was first proposed by Rowland and Perrett^58^, but it has only rarely been adopted in studies of non-human primates. Such an approach will enable us to investigate how each of facial features affects non-human primates’ facial recognition with various dimensions. Although the current study suggests that facial coloration is a critical element of age categorisation in chimpanzees, we do not know whether it also plays an important role in other general attributes. In humans, how much shape and colour information contribute to face perception varies across facial attributes to be judged. For example, past studies have shown that facial shape plays a more important role than colour in determining notions of ‘female dominance’ and ‘identity’, but the opposite is true for ‘male attractiveness’ and ‘perceived age’^31,59^. Future studies should address whether chimpanzees and other non-human primates rely more on facial shape information rather than colour when judging other aspects of faces such as sex, attractiveness, or identity. Of course, this approach is not limited to chimpanzees, but applicable for other non-human primates. For example, it is known that both facial redness and symmetry contribute attractiveness in rhesus macaques^33,60^, but it has been unclear which is more important for them. Using a morphing technique as we did in this study will deepen our knowledge on face processing in non-human primates.

## Supplementary information

Supplementary Information 1.

Supplementary Datasets.

## Data Availability

The datasets generated or analysed during this study are included in this published article (and its Supplementary Information files).
