# Supplementary material for: Colour matters more than shape for chimpanzees’ recognition of developmental face changes
Source: Sci Rep. 2020 Oct 23;10:18201. doi: 10.1038/s41598-020-75284-2 (PMC7584574; doi:10.1038/s41598-020-75284-2)
Supplement: Supplementary file 1 — Supplementary Information 1. [file 41598_2020_75284_MOESM1_ESM.docx]

**Colour matters more than shape for chimpanzees’ recognition of developmental face changes**

# Yuri Kawaguchi^1,2*^, Koyo Nakamura^2, 3, 4^, Masaki Tomonaga^1^

# Supplementary Note

During the training, three chimpanzees (Cleo, Pendesa and Ayumu) dropped out because their performance did not sufficiently improve after 15 consecutive ‘block-sessions’ when they were trained to discriminate the first stimulus pair. In a block-session, one session was divided into two to six blocks and the same sample stimulus was presented within one block. We started with a two-block session in which one stimulus was presented in the first half trials and the other stimulus was presented in second half trials in one session. If their performance achieved the criterion (more than 85 % in two consecutive sessions), we conducted the next block session where one session was divided into more blocks (i.e. a four-block session or six-block session) which means the same stimulus presented fewer consecutive trials.

# Supplementary Figure


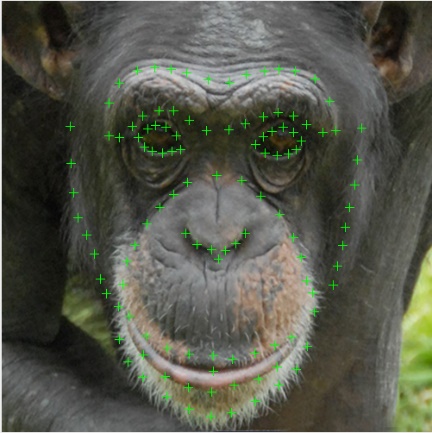


Figure S1: An example of facial landmarks. The landmarks were placed with Psychomorph software (version 6; https://users.aber.ac.uk/bpt/jpsychomorph/).


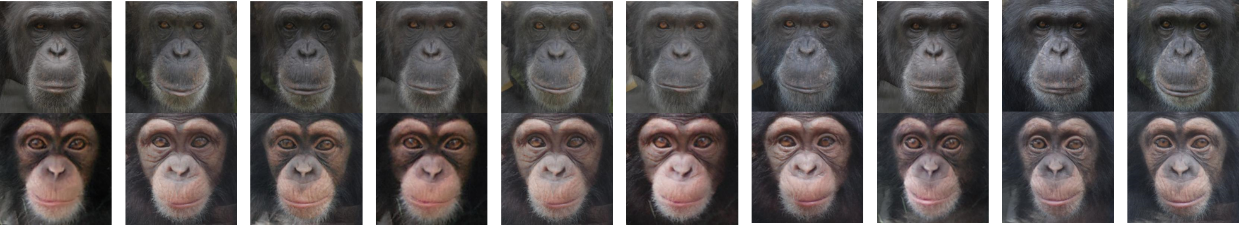


Figure S2: The 10 pairs of three-morphed average faces used for training and image analysis. The facial images were generated with Psychomorph software (version 6; https://users.aber.ac.uk/bpt/jpsychomorph/).


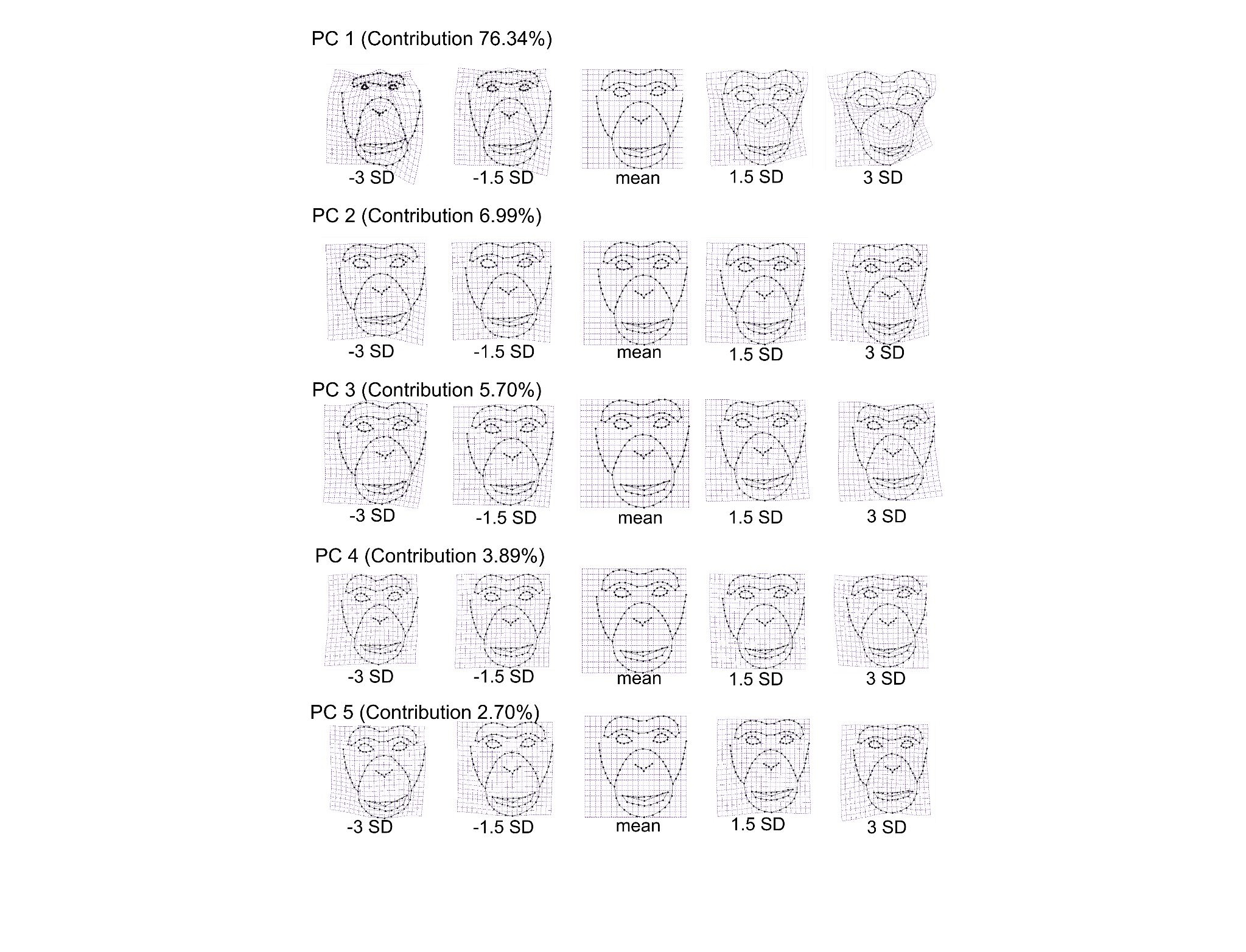


Figure S3. Facial shape variations along the axis of the principal components. These first five components accounted for more than 95% of facial shape variation in total: The theoretical values of -3, -1.5 SD, average, +1.5, +3 SD. The facial images were generated with TpsRelw software (version 1.70; https://tpsrelw.software.informer.com/).


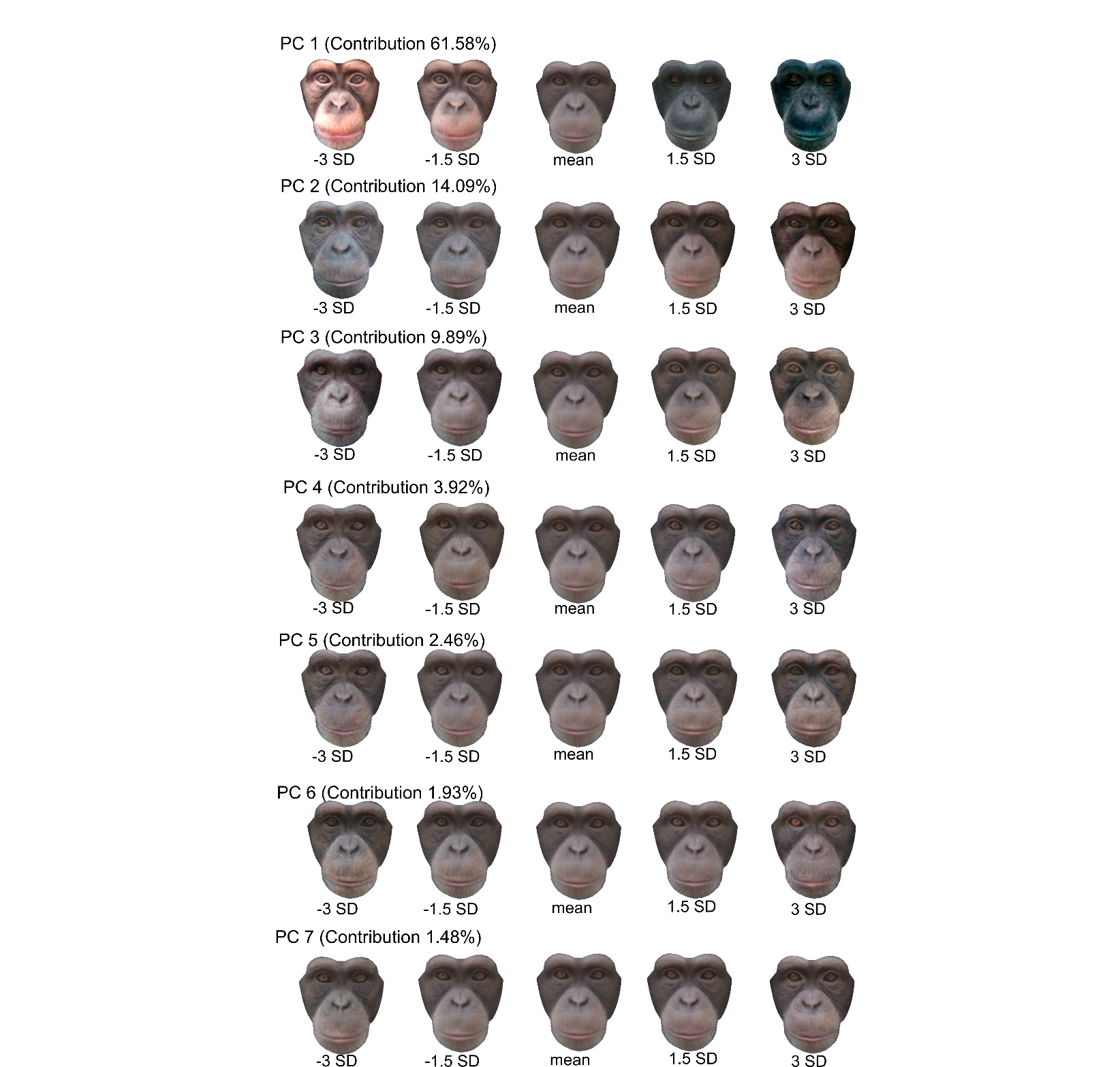


Figure S4. Facial colour variations along the axis of the principal components. These first seven components accounted for more than 95% of facial shape variation in total: The theoretical values of -3, -1.5 SD, average, +1.5, +3 SD. The facial images were generated with MATLAB (version 2018a; https://uk.mathworks.com/products/matlab.html).

Table S1 The order of the stimuli pairs used in the training phase

|  | Ai | Ayumu | Chloe | Cleo | Pal | Pen |
| --- | --- | --- | --- | --- | --- | --- |
| Step 1 | pair 1 | pair 1 | pair 7 | pair 7 | pair 4 | pair 4 |
| Step 2 | pair 2, 3, 4 | ‐ | pair 2, 4, 10 | ‐ | pair 1, 2, 10 | ‐ |
| Step 3 | pair 5, 6, 7 | ‐ | pair 5, 6, 8 | ‐ | pair 3, 5, 7 | ‐ |
| Step 4 | pair 8, 9, 10 | ‐ | pair 1, 3, 9 | ‐ | pair 6, 8, 9 | ‐ |
